# Tocilizumab for treating severe COVID‐19 pneumonia refractory to combined hydroxychloroquine, lopinavir plus ritonavir, and favipiravir: A case series

**DOI:** 10.1002/ccr3.3407

**Published:** 2020-10-13

**Authors:** Manoon Surabotsophon, Yingyot Klai‐On, Vipa Thanachartwet, Sirote Khunapornphairote, Supat Chamnanchanunt, Theerapat Racharak, Wannada Laisuan, Duangjai Sahassananda, Samlee Hapunna, Somjit Jinapuk, Suthee Leelasetakul, Varunee Desakorn

**Affiliations:** ^1^ Pulmonary and Critical Care Division Department of Medicine Ramkhamhaeng Hospital Bangkok Thailand; ^2^ Department of Clinical Tropical Medicine Faculty of Tropical Medicine Mahidol University Bangkok Thailand; ^3^ Nephrology Unit Department of Medicine Ramkhamhaeng Hospital Bangkok Thailand; ^4^ Heart Center Department of Medicine Ramkhamhaeng Hospital Bangkok Thailand; ^5^ Division of Allergy Immunology and Rheumatology Department of Medicine Faculty of Medicine Ramathibodi Hospital Mahidol University Bangkok Thailand; ^6^ Information Technology Unit Faculty of Tropical Medicine Mahidol University Bangkok Thailand; ^7^ Intensive Care Unit Ramkhamhaeng Hospital Bangkok Thailand; ^8^ Central Laboratory Centre Ramkhamhaeng Hospital Bangkok Thailand; ^9^ Division of Oncology Department of Medicine Ramkhamhaeng Hospital Bangkok Thailand

**Keywords:** COVID‐19, pneumonia, acute respiratory distress syndrome, tocilizumab

## Abstract

Three patients diagnosed with severe COVID‐19 pneumonia received treatment with hydroxychloroquine combined with lopinavir, ritonavir, and favipiravir. Two patients diagnosed early, received tocilizumab when the pneumonia became severe and survived. The thrid patient was diagnosed late and received tocilizumab when the disease progressed to acute respiratory distress syndrome, and died.

## Graphical abstract

Tocilizumab could be used in patients with severe COVID‐19 pneumonia who did not show response to a combination of antiviral therapy. However, the use of a combination antiviral therapy should be further confirmed in clinical trials.

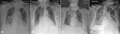

## 1 INTRODUCTION

Coronavirus disease 2019 (COVID‐19), caused by the severe acute respiratory syndrome coronavirus 2 (SARS‐CoV‐2), was first reported as the cause of bilateral interstitial pneumonia in Wuhan, Hubei province, China, in January 2020.^1^ COVID‐19 has spread rapidly worldwide being highly contagious. In March 2020, the World Health Organization (WHO) declared COVID‐19 as a global pandemic.^2^, ^3^

Previous reports have shown common symptoms among patients with COVID‐19 including fever (89%), dry cough (68%), fatigue (38%), and dyspnoea (19%).^4^ Most patients with COVID‐19 (84%) have mild disease and spontaneously recover, and the rest (16%) develop severe disease.^4^ The most common manifestation of these patients is pneumonia, which may rapidly progress to severe pneumonia and then to acute respiratory distress syndrome (ARDS) and/or organs dysfunction. This is potentially due to an excessive host immune response by hyperactivation of cytotoxic T cells and humoral immune response, particularly of interleukin (IL)‐6, which plays an important role as a primary critical mediator for the development of cytokine storms, leading to respiratory failure, shock, and organ dysfunction.^4^, ^5^, ^6^

A previous study demonstrated a mortality rate of 8.1% among patients with severe COVID‐19 pneumonia, and only 0.1% of them had nonsevere COVID‐19.^4^ Currently, there is no proven effective treatment for patients with severe COVID‐19 pneumonia. A recent randomized controlled trial on lopinavir plus ritonavir revealed no difference in time to clinical improvement and mortality between adults hospitalized with severe COVID‐19 pneumonia and those who received standard care.^7^ Similarly, a systemic review showed that chloroquine or hydroxychloroquine could not reduce mortality compared with standard care among hospitalized patients with COVID‐19.^8^ However, a previous nonrandomized controlled trial revealed favipiravir as an independent factor associated with faster viral clearance.^9^ In Thailand, national regulations have recommended a combination antiviral therapy, including chloroquine or hydroxychloroquine and darunavir plus ritonavir or lopinavir plus ritonavir combined favipiravir, for 10 days in patients with COVID‐19 pneumonia. This may inhibit viral replication enabling the progression of the disease to a hyperinflammatory state.^10^ Thereafter, patients should be monitored for clinical findings, including the ratio of arterial oxygen saturation (SpO_2_) and fraction of inspired oxygen (FiO_2_), chest radiograph, high sensitivity C‐reactive protein (hs‐CRP), and lactate dehydrogenase (LDH). In cases of severe COVID‐19 pneumonia, IL‐6 levels should be monitored.

In addition, a systematic review indicated that an anti‐IL‐6 receptor antibody may benefit patients with severe COVID‐19 pneumonia, although the effectiveness of anti‐IL‐6 receptor antibody, such as tocilizumab for COVID‐19 remains uncertain.^11^ The early recognition of SARS‐CoV‐2 infection and the timely treatment of COVID‐19 patients with particularly severe pneumonia are important for preventing the occurrence of cytokine storms and ensuring patient survival.^6^, ^12^

Herein, we report three patients with confirmed SARS‐CoV‐2 infection who developed severe pneumonia that was refractory to treatment combination of hydroxychloroquine, lopinavir plus ritonavir, and favipiravir. Tocilizumab was then administered, and the disease severity was monitored using IL‐6 and hs‐CRP levels.

## 2 CASE HISTORY

### 2.1 Case 1

A 58‐year‐old Thai man with a body weight of 70 kg, height of 175 cm, body mass index (BMI) of 22.8 kg/m^2^, who had chronic medical illness of hypertension, hyperlipidemia, and coronary artery disease presented to the emergency department with symptoms of fever with chills, productive cough with white phlegm, sneezing, sore throat, and myalgia. The patient received hydroxychloroquine, lopinavir plus ritonavir, and favipiravir for 10 days combined with azithromycin for 5 days. His recent medications included manidipine (10 mg once daily, orally), atenolol (25 mg once daily, orally), rosuvastatin (20 mg once daily, orally), clopidogrel (75 mg once daily, orally), and aspirin (81 mg once daily, orally). He was a healthcare worker who had been in contact with a hospitalized patient with confirmed SARS‐CoV‐2 infection on day ‐17 of hospitalization and was quarantined thereafter. On day ‐3 of hospitalization, both samples of nasopharyngeal swab and oropharyngeal swab showed undetectable levels of SARS‐CoV‐2 RNA using real‐time reverse transcription‐polymerase chain reaction (RT‐PCR), and the patient showed no symptoms.

**Figure 1:**
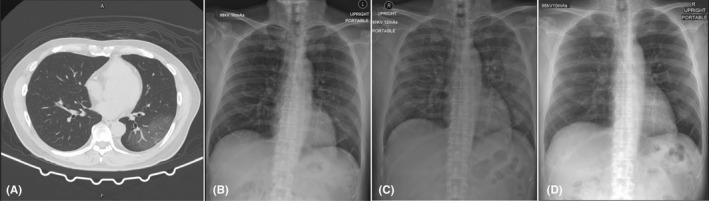
Chest imaging of a 58‐year‐old Thai man (case 1). A, Chest computed tomography scan on day of hospitalization, showing ground‐glass infiltration at the posterior segment of the left lower lobe by air bronchogram. B, Chest radiograph on day 2 of hospitalization, showing progression of interstitial infiltration in the right middle and left lower lung zones. C, Chest radiograph on day 8 of hospitalization, showing new alveolar infiltration in the right upper lung zone and increased alveolar infiltration in the right middle, left upper, and left lower lung zones. D, Chest radiograph on day 27 of hospitalization was normal

On day ‐2 of hospitalization, he had fever with chills, productive cough with white phlegm, sneezing, sore throat, and myalgia. The patient received treatment with oseltamivir (150 mg twice daily, orally) and azithromycin (500 mg once daily, orally), but the symptoms did not improve. On the day of hospitalization, body temperature 37.9 ºC, heart rate 90 beats/min, blood pressure 136/85 mmHg, respiratory rate 20 breaths/min, and SpO_2_ 97% in room air (Table 1). Chest radiograph was normal, but chest computed tomography (CT) showed ground‐glass infiltration at the posterior segment of the left lower lobe by air bronchogram (Figure 1A). RT‐PCR assay showed detectable levels of SARS‐CoV‐2 RNA at the region of ORF1ab/RdRp, E and N gene, in both samples of nasopharyngeal swab and throat swabs. Laboratory findings showed low absolute lymphocyte count (ALC; 419 cells/mm^3^). The patient was admitted to the negative pressure isolation ward and received treatment of hydroxychloroquine (400 mg every 12 hours for 1 day, then 200 mg every 12 hours, orally), darunavir plus ritonavir (800/100 mg once daily, orally) as antiviral treatment, starting on day‐ 1 of hospitalization. Azithromycin (500 mg once daily, orally) was administered as an anti‐microbial and anti‐inflammatory agent (Table 1).

**Table 1:** Symptoms and signs, laboratory findings, and management of a 58‐year‐old Thai man (case 1) from day of hospitalization to day 15 of hospitalization

Day of hospitalization	0 (Ward)	1 (Ward)	2 (Ward)	3 (Ward)	4 (Ward)	5 (Ward)	6 (Ward)	7 (Ward)	8 (ICU)	9 (ICU)	10 (ICU)	11 (Ward)	12 (Ward)	13 (Ward)	14 (Ward)	15 (Ward)
Symptoms and signs																
Fever (ºC)	37.9	38.3	37.1	37.5	37.9	37.9	38.7	38.5	38.4	37.1	36.8	37.0	37.0	37.0	36.5	36.6
Cough	++	++	++	++	++	++	+++	+++	+++	++	+	+	+	+	−	−
Sneezing	+	+	−	−	−	−	−	−	−	−	−	−	−	−	−	−
Sore throat	++	+	−	−	−	−	−	−	−	−	−	−	−	−	−	−
Myalgia	+	−	−	−	−	−	−	−	−	−	−	−	−	−	−	−
Fatigue	−	−	−	+	+	++	++	+++	+++	++	+	+	−	−	−	−
Anorexia	−	−	−	−	+	++	++	+++	+++	+++	+	+	−	−	−	−
Diarrhea	−	−	−	+	++	++	+	+	+	++	+	+	−	−	−	−
SOB	−	−	−	+	+	++	+++	+++	+++	++	+	−	−	−	−	−
RR (breaths/min)	20	22	20	22	20	20	22	24	28	26	26	26	22	20	18	18
SpO_2_/FiO_2_ (ratio)	97/0.21 (462)	95/0.21 (452)	95/0.21 (452)	92/0.21 (438)	90/0.21 (428)	92/0.21 (438)	88/0.21 (419)	88/0.21 (419)	88/0.21 (419)	91/0.21 (433)	93/0.21 (443)	94/0.21 (448)	96/0.21 (457)	96/0.21 (457)	95/0.21 (452)	97/0.21 (462)
HR (beats/min)	90	86	88	88	80	88	104	94	92	90	90	94	90	94	94	90
Laboratory findings																
SARS‐CoV‐2 RNA	detected	−	−	−	−	−	−	−	−	−	−	−	−	detected	−	−
Hgb (g/dL)	14.3	−	15.2	−	−	−	−	−	14.3	13.8	14.6	15.6	−	−	15.0	−
Hct (%)	41.3	−	43.9	−	−	−	−	−	40.7	40.0	42.2	45.5	−	−	42.7	−
WBC (cells/mm^3^)	6160	−	4510	−	−	−	−	−	10460	7310	4740	4740	−	−	7610	−
ANC (cells/mm^3^)	5347	−	3324	−	−	−	−	−	9749	6667	4090	3266	−	−	5350	−
ALC (cells/mm^3^)	419	−	708	−	−	−	−	−	408	351	289	758	−	−	1088	−
Plts (/mm^3^)	258000	−	251000	−	−	−	−	−	393000	411000	495000	540000	−	−	597000	−
hs‐CRP (mg/L)	−	−	−	−	−	−	−	−	164.7	155.8	63.1	31.6	−	−	2.9	−
IL‐6 (pg/mL)	−	−	−	−	−	−	−	−	46.6	784.0	−	−	−	−	−	−
ALT (U/L)	28	−	−	−	−	−	−	−	36	41	−	56	−	−	−	−
LDH (U/L)	−	−	−	−	−	−	−	−	409	−	−	−	−	−	−	−
Cr (mg/dlL)	1.1	−	−	−	−	−	−	−	0.9	−						−
Management																
Oxygen (L/min)	−	−	−	NC (5)	NC (5)	NC (5)	NC (5)	NC (5)	NC (5)	NC (5)	NC (5)	NC (5)	NC (3)	NC (3)	NC (3)	−
Hydroxychloroquine	−	400 mg q 12 h	200 mg q 12 h	200 mg q 12 h	200 mg q 12 h	200 mg q 12 h	200 mg q 12 h	200 mg q 12 h	200 mg q 12 h	200 mg q 12 h	200 mg q 12 h	−	−	−	−	−
Darunavir + Ritonavir	−	800/100 mg	800/100 mg	−	−	−	−	−	−	−	−	−	−	−	−	−
Lopinavir + Ritonavir	−	−	−	400/100 mg q 12 h	400/100 mg q 12 h	400/100 mg q 12 h	400/100 mg q 12 h	400/100 mg q 12 h	400/100 mg q 12 h	400/100 mg q 12 h	400/100 mg q 12 h	400/100 mg q 12 h	400/100 mg q 12 h	−	−	−
Favipiravir	−	−	−	1600 mg q 12 h	600 mg q 12 h	600 mg q 12 h	600 mg q 12 h	600 mg q 12 h	600 mg q 12 h	600 mg q 12 h	600 mg q 12 h	600 mg q 12 h	600 mg q 12 h	−	−	−
Tocilizumab	−	−	−	−	−	−	−	−	560 mg	560 mg	−	−	−	−	−	−
Azithromycin	−	500 mg	500 mg	500 mg	500 mg	500 mg	−	−	−	−	−	−	−	−	−	−

Abbreviations: ALC, absolute lymphocyte count; ALT, alanine aminotransferase; ANC, absolute neutrophil count; Cr, creatinine; FiO_2_, fraction of inspired oxygen; Hct, hematocrit; Hgb, hemoglobin; HR, heart rate; hs‐CRP, high sensitivity C‐reactive protein; ICU, intensive care unit; IL‐6, interleukin‐6; LDH, lactate dehydrogenase; NC, nasal cannula; Plts, platelets; RR, respiratory rate; SOB, shortness of breath; SpO_2_, arterial oxygen saturation; WBC, white blood cell count.

Chest radiograph on day‐ 2 of hospitalization showed progression of interstitial infiltration in the right middle and left lower lung zones (Figure 1B). On day‐ 3 of hospitalization, the patient developed symptoms of fatigue, watery diarrhea, shortness of breath, and hypoxemia (SpO_2_ 92% in room air; Table 1). Darunavir plus ritonavir were then stopped, and lopinavir plus ritonavir (400/100 mg every 12 hours, orally) combined with favipiravir (1600 mg every 12 hours for 1 day, then 600 mg every 12 hours, orally) were prescribed as antiviral treatment (Table 1).

On day‐ 8 of hospitalization (Table 1), the patient still had fever with productive cough, fatigue, anorexia, watery diarrhea, shortness of breath, and hypoxemia (SpO_2_ 88% in room air). He was then transferred to the intensive care unit (ICU) with negative pressure isolation. Chest radiograph showed new alveolar infiltration in the right upper lung zone and increased alveolar infiltration in the right middle, left upper, and left lower lung zones (Figure 1C). Laboratory findings showed low ALC (408 cells/mm^3^), increased hs‐CRP (164.7 mg/L), increased levels of IL‐6 (46.6 pg/mL), and increased levels of LDH (409 U/L). Tocilizumab (8 mg/kg/dose intravenous drip for 90 minutes with repeated dose in the next 12 hours) was administered due to progression of hypoxemia and increased lung infiltration. The patient also received supplement oxygen of 5 L/min via a nasal cannula in the prone position to keep SpO_2_ >94%.

On day‐ 9 of hospitalization (Table 1), the IL‐6 levels prior to receiving the second dose of tocilizumab markedly increased to 784.0 pg/mL. The patient’s symptoms improved, with no fever, decreased cough, decreased shortness of breath, and increased SpO_2_ (91% in room air). Laboratory findings showed low ALC (351 cells/mm^3^), decreased hs‐CRP levels (155.8 mg/L), and mildly elevated alanine aminotransferase (ALT) levels (41 U/L). Chest radiograph showed slightly decreased infiltration in both upper lung zones, but infiltration in both lower lung zones was unchanged. On day‐ 10 of hospitalization (Table 1), the symptoms gradually improved. Chest radiograph showed decreased lung infiltration, and laboratory findings showed low ALC (289 cells/mm^3^) and markedly decreased levels of hs‐CRP (63.1 mg/L).

On day‐ 11 of hospitalization (Table 1), laboratory findings showed increased ALC (758 cells/mm^3^), decreased hs‐CRP levels (31.6 mg/L), and mildly elevated ALT levels (56 U/L). Chest radiograph showed markedly decreased infiltration on day‐ 18 of hospitalization and was normal on day‐ 27 of hospitalization (Figure 1D). However, both samples of nasopharyngeal and oropharyngeal swabs still showed detectable levels of SARS‐CoV‐2 RNA using RT‐PCR on day‐ 36 of hospitalization, although the patient had no symptoms.

### 2.2 Case 2

A 34‐year‐old Thai woman with a body weight of 74.6 kg, height of 160 cm, and BMI of 29.1 kg/m^2^, presented to an acute respiratory infection clinic with symptoms of fever (36.2°C‐38.6°C) and sore throat. Physical examination revealed enlargement of both tonsils with exudate. She was diagnosed with acute exudative tonsillitis and received treatment with amoxicillin plus clavulanate (1 g every 12 hours, orally). She was a healthcare worker and had had contact with a hospitalized patient with confirmed SARS‐CoV‐2 infection on day ‐18 of hospitalization and was thereafter quarantined. On day ‐4 of hospitalization, both samples of nasopharyngeal swab and throat swab showed undetectable levels of SARS‐CoV‐2 RNA, and the patient had no symptoms. Both samples of nasopharyngeal and oropharyngeal swabs were retested for SARS‐CoV‐2 RNA on day ‐1 of hospitalization. The results showed detectable SARS‐CoV‐2 RNA levels in the region of ORF1ab, N gene, NS‐1, and NS‐2 in both samples (Table 2).

**Table 2:** Symptoms and signs, laboratory findings, and management of a 34‐year‐old Thai woman (case 2) from the day ‐1 of hospitalization to day 16 of hospitalization

Day of hospitalization	‐1 (ARI clinic)	0 (Ward)	1 (Ward)	2 (Ward)	3 (Ward)	4 (Ward)	5 (Ward)	6 (ICU)	7 (ICU)	8 (ICU)	9 (ICU)	10 (ICU)	11 (ICU)	12 (Ward)	13 (Ward)	14 (Ward)	15 (Ward)	16 (Ward)
Symptoms and signs																		
Fever (ºC)	38.6	37.0	38.1	38.8	38.5	38.3	39.1	38.7	37.1	36.8	35.9	36.8	36.1	36.3	36.7	36.3	36.2	36.2
Cough	−	+	+	+	+	+	++	+++	+++	+++	+++	++	++	++	+	+	−	−
Sore throat	++	++	+	+	+	−	−	−	−	−	−	−	−	−	−	−	−	−
Myalgia	−	++	++	++	++	++	++	++	++	++	+	−	−	−	−	−	−	−
Fatigue	−	+	+	+	+	++	+++	+++	++	+	−	−	−	−	−	−	−	−
Anorexia	−	−	+++	+++	+++	+++	+++	+++	++	++	++	+	−	−	−	−	−	−
Vomiting	−	−	+++	+++	+++	+++	+++	+++	++	++	+	+	−	−	−	−	−	−
Diarrhea	−	−	−	−	+	+	++	++	+++	+	−	−	−	−	−	−	−	−
Pleurisy	−	−	−	−	−	−	+	+++	++	+	−	−	−	−	−	−	−	−
SOB	−	−	−	−	−	−	+	+++	++	++	++	++	++	+	−	−	−	−
Rash	−	−	−	−	−	−	−	−	−	−	−	++	+++	++	+	+	+	+
RR (breaths/min)	−	18	20	22	22	24	26	40	34	32	28	24	24	22	18	16	18	18
SpO_2_/FiO_2_ (ratio)	−	96/0.21 (457)	96/0.21 (457)	95/0.21 (452)	95/0.21 (452)	95/0.21 (452)	95/0.21 (452)	87/0.21 (414)	91/0.21 (433)	90/0.21 (428)	90/0.21 (428)	91/0.21 (433)	90/0.21 (428)	92/0.21 (438)	96/0.21 (457)	96/0.21 (457)	95/0.21 (452)	97/0.21 (462)
HR (beats/min)	−	106	102	116	110	104	120	122	100	92	82	82	82	82	78	72	68	68
Laboratory findings																		
SARS‐CoV‐2 RNA	detected	−	−	−	−	detected	−	−	−	−	−	−	−	−	detected	−	−	undetected
Hgb (g/dL)	−	13.9	−	−	−	−	14.6	14.4	13.8	−	14.7	−	15.2	−	−	−	−	13.8
Hct (%)	−	41.7	−	−	−	−	43.3	42.5	40.8	−	44.5	−	46.2	−	−	−	−	41.0
WBC (cells/mm^3^)	−	7880	−	−	−	−	6580	8980	5630	−	2490	−	3130	−	−	−	−	3040
ANC (cells/mm^3^)	−	5752	−	−	−	−	4988	7408	4448	−	946	−	1240	−	−	−	−	1094
ALC (cells/mm^3^)	−	1592	−	−	−	−	1309	1158	1013	−	1370	−	1440	−	−	−	−	1368
Plts (/mm^3^)	−	203000	−	−	−	−	237000	299000	307000	−	373000	−	448000	−	−	−	−	322000
hs‐CRP (mg/L)	−	89.6	−	−	−	−	74.3	145.4	120.8	−	5.4	−	1.3	−	−	−	−	0.6
IL‐6 (pg/mL)	−	−	−	−	−	−	−	49.8	865.0	−	−	−	−	−	−	−	−	
ALT (U/L)	−	19	−	−	−	−	27	−	−	−	−	−	115	−	−	−	−	303
LDH (U/L)	−	−	−	−	−	−	−	380	369	−	−	−	323	−	−	−	−	345
Cr (mg/dL)	−	0.6	−	−	−	−	0.7	−	0.6	−	−	−	0.9	−	−	−	−	0.6
Management																		
Oxygen (L/min)	−	−	−	−	−	−	−	FM (10)	NC (5)	NC (5)	NC (3)	NC (3)	NC (3)	NC (3)	NC (3)	−	−	−
Hydroxychloroquine	−	400 mg q 12 h	200 mg q 12 h	200 mg q 12 h	200 mg q 12 h	200 mg q 12 h	200 mg q 12 h	200 mg q 12 h	200 mg q 12 h	200 mg q 12 h	200 mg q 12 h	−	−	−	−	−	−	−
Lopinavir + Ritonavir	−	400/100 mg q 12 h	400/100 mg q 12 h	400/100 mg q 12 h	400/100 mg q 12 h	400/100 mg q 12 h	400/100 mg q 12 h	400/100 mg q 12 h	400/100 mg q 12 h	400/100 mg q 12 h	400/100 mg q 12 h	−	−	−	−	−	−	−
Favipiravir	−	−	−	1600 mg q 12 h	600 mg q 12 h	600 mg q 12 h	600 mg q 12 h	600 mg q 12 h	600 mg q 12 h	600 mg q 12 h	600 mg q 12 h	600 mg q 12 h	600 mg q 12 h	600 mg q 12 h	600 mg q 12 h	600 mg q 12 h	600 mg q 12 h	−
Tocilizumab	−	−	−	−	−	−	−	560 mg	560 mg	−	−	−	−	−	−	−	−	−
Azithromycin	−	500 mg	250 mg	250 mg	250 mg	250 mg	250 mg	250 mg	250 mg	250 mg	250 mg	250 mg	250 mg	250 mg	250 mg	−	−	−

Abbreviations: ARI, acute respiratory infection; ALT, alanine aminotransferase; ALC, absolute lymphocyte count; ANC, absolute neutrophil count; Cr, creatinine; FiO2, fraction of inspired oxygen; FM, face mask; Hgb, hemoglobin; Hct, hematocrit; HR, heart rate; hs‐CRP, high sensitivity C‐reactive protein; ICU, intensive care unit; IL‐6, interleukin‐6; LDH, lactate dehydrogenase; NC, nasal cannula; Plts, platelets; RR, respiratory rate; SpO2, arterial oxygen saturation; SOB, shortness of breath; WBC, white blood cell count.

**Figure 2:**
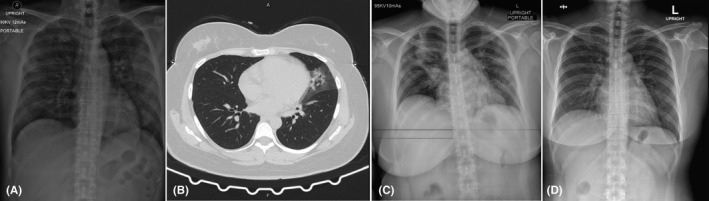
Chest imaging of a 34‐year‐old Thai woman (case 2). A, Chest radiograph on day of hospitalization, showing minimal interstitial infiltration in the left lower lung zone. B, Chest computed tomography scan without contrast on day of hospitalization, showing patchy ground glass, with overlying consolidation opacity, and interlobar septal thickening in the inferior lingular segment of the left upper lobe. C, Chest radiograph on day 6 of hospitalization, showing progression of alveolar infiltration in the right upper and middle lung zones and increased interstitial infiltration in the left upper, middle, and left lower lung zones. D, Chest radiograph on day 36 of hospitalization was normal

On the day of hospitalization (Table 2), the patient had fever, dry cough, sore throat, myalgia, and fatigue. She received antiviral treatment with hydroxychloroquine and lopinavir plus ritonavir for 10 days, combined with favipiravir and azithromycin for 14 days. A physical examinations revealed body temperature 37.0°C, heart rate 106 beats/min with regular rate and rhythm, blood pressure 120/80 mmHg, respiratory rate 18 breaths/min, and SpO_2_ 96% in room air. Both tonsils were still enlarged with exudate. Chest radiograph showed minimal interstitial infiltration in the left lower lung zone (Figure 2A), and chest CT scan without contrast showed patchy ground glass with overlying consolidation opacity and interlobar septal thickening in the inferior lingular segment of the left upper lobe (Figure 2B). Laboratory findings showed ALC of 1592 cells/mm^3^.

The patient was admitted to the negative pressure isolation ward and received antiviral treatment with hydroxychloroquine (400 mg every 12 hours for 1 day, then 200 mg every 12 hours, orally) and lopinavir plus ritonavir (400/100 mg every 12 hours, orally) combined with azithromycin (500 mg, then 250 mg, once daily, orally) for anti‐inflammation. Amoxicillin plus clavulanate (1.2 g every 12 hours, intravenously) was given for continued treatment of acute exudative tonsillitis. Favipiravir (1600 mg every 12 hours for 1 day, then 600 mg every 12 hours, orally) was added as an antiviral treatment on day‐ 2 of hospitalization, after awaiting drug delivery from the hospital of Ministry of Public Health in Thailand.

On day‐ 5 of hospitalization (Table 2), her fever rose to 39.1ºC combined with increased symptoms of cough, myalgia, fatigue, anorexia, vomiting, watery diarrhea, pleuritic chest pain, and shortness of breath, but no sore throat and no enlargement of both tonsils. Amoxicillin plus clavulanate was then stopped and cefoperazone plus sulbactam (1.5 g every 12 hours, intravenously) was administered due to suspected nosocomially acquired infections. Laboratory findings showed low ALC (1309 cells/mm^3^).

On day‐ 6 of hospitalization, chest radiograph showed progression of alveolar infiltration at the right upper and right middle lung zone and increased interstitial infiltration in the left upper, middle, and lower lung zone (Figure 2C). The patient was then transferred to the ICU with negative pressure isolation due to decreased SpO_2_ (87%) in room air and increased shortness of breath with a respiratory rate of 40 breaths/min. The patient received supplement oxygen via a face mask in the prone position in order to keep SpO_2_ >94%. Tocilizumab (8 mg/kg/dose intravenous drip for 90 min with repeated dose for the next 12 hours) was then administered to attenuate lung inflammation, as hypoxemia developed. Laboratory findings showed low ALC (1158 cells/mm^3^) and increased levels of hs‐CRP (145.4 mg/L), IL‐6 (49.8 pg/mL), and LDH (380 U/L) (Table 2).

On day‐ 7 of hospitalization, the symptoms showed improvement, the body temperature dropped (37.1 ºC), and SpO_2_ was increased (91% in room air). Laboratory findings showed low ALC (1013 cells/mm^3^), decreased levels of hs‐CRP (120.8 mg/L) and LDH (369 U/L), and increased levels of IL‐6 (865.0 pg/mL) (Table 2). On day‐ 8 of hospitalization, chest radiograph showed decreased infiltration in both lungs, but new alveolar infiltration was observed in the right lower lung zone. On day‐ 9 of hospitalization, laboratory findings showed increased ALC (1370 cells/mm^3^) and markedly decreased hs‐CRP levels (5.4 mg/L) (Table 2).

**Figure 3:**
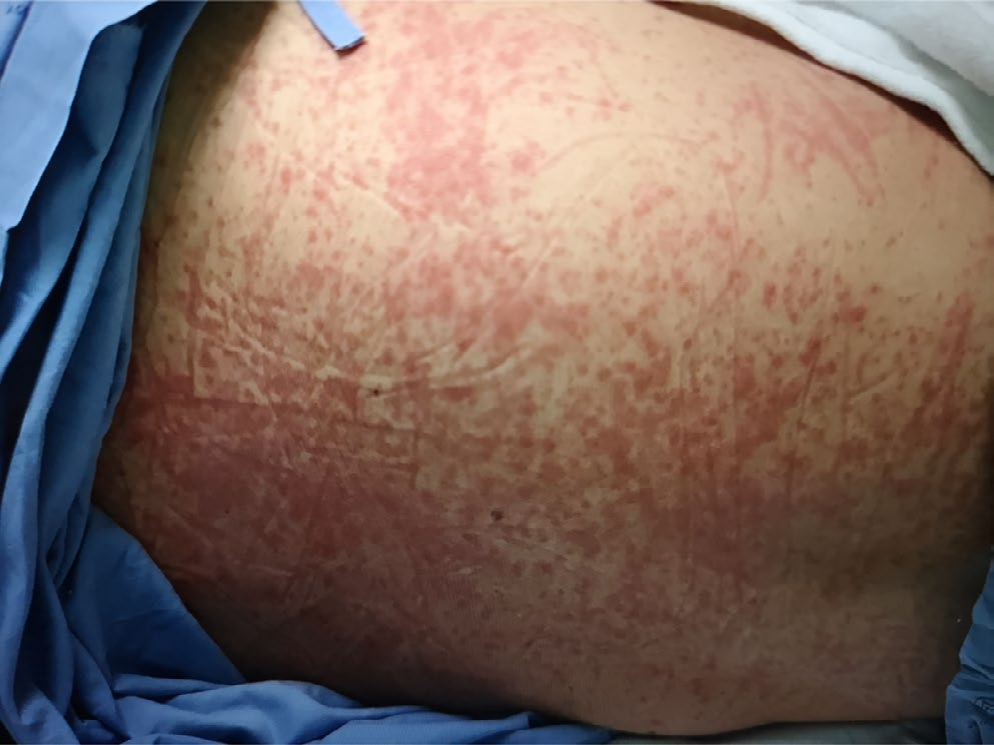
Generalized maculopapular rash throughout the face, neck, and trunk in a 34‐year‐old Thai woman (case 2) observed on day 12 of hospitalization

On day‐ 10 of hospitalization, a maculopapular rash with itching was observed on both arms and thighs. The patient received antihistamine medications, including chlorpheniramine (10 mg every 8 hours, intravenously), diphenhydramine (50 mg once a day, orally), and cetirizine (20 mg every 12 hours, orally). Generalized maculopapular rash was then found to have spread throughout the face, neck, and trunk on day‐ 12 of hospitalization (Figure 3). The rash decreased on day‐ 13 of hospitalization, 3 days after stopping hydroxychloroquine and lopinavir plus ritonavir (Table 2).

On day‐ 11 of hospitalization, ALT levels (115 U/L) were elevated and rose to 303 U/L on day‐ 16 of hospitalization (Table 2), but decreased to 170 U/L on day‐ 36 of hospitalization.

On day‐ 12 of hospitalization, chest radiograph showed mark decreased infiltration in both lungs, and SpO_2_ increased (96% in room air) on day‐ 13 of hospitalization (Table 2). Hemocultures and sputum culture were negative. The patient was transferred back to the negative pressure isolation ward.

Chest radiograph was normal on day‐ 36 of hospitalization (Figure 2D). RT‐PCR assay for the detection of SARS‐CoV‐2 RNA was negative on two repeated samples of nasopharyngeal and oropharyngeal swabs day‐ 16 of hospitalization and on day‐ 33 of hospitalization.

### 2.3 Case 3

A 78‐year‐old Thai woman presented to the emergency department with right hemiparesis and drowsiness after a fall due to acute left thalamic infarction with intraventricular hemorrhage. The patient died on day‐ 31 of hospitalization due to septic shock. Urine culture showed >10^5^ CFU/mL of *Candida albicans* and *Stenotrophomonas maltophilia*. She had underlying chronic medical illness, including hypertension, hyperlipidemia, and paroxysmal atrial fibrillation. Physical examination showed Glasgow Coma Scale (GCS) score of 8 (E3M1V4), blood pressure of 187/84 mm Hg, and motor power grade II on the right extremities. The patient received normal saline infusion, and blood pressure was controlled in the ICU. The GCS score increased to 12 (E4M2V6), and motor power increased to grade III on the right extremities within 24 hours after treatment (Table 3).

**Table 3:** Symptoms and signs, laboratory findings, and management of a 78‐year‐old Thai woman (case 3) from the day of hospitalization to day 15 of hospitalization

Day of hospitalization	0 (ICU)	1 (ICU)	2 (ICU)	3 (ICU)	4 (ICU)	5 (ICU)	6 (Ward)	7 (Ward)	8 (Ward)	9 (Ward)	10 (Ward)	11 (Ward)	12 (Ward)	13 (Ward)	14 (Ward)	15 (Ward)
Symptoms and signs																
Fever (ºC)	37.2	36.5	36.4	38.4	37.5	36.9	36.6	36.8	36.7	36.6	37.0	36.6	36.3	37.0	37.2	37.2
Cough	−	−	−	−	−	−	−	−	−	−	−	−	−	+	+	+
Fatigue	−	−	−	−	−	−	−	−	−	−	+	+	+	+	+	+
Diarrhea	−	−	−	−	−	−	−	−	−	−	++	+	+	+	+	+
SOB	−	−	−	−	−	−	−	−	−	−	−	−	−	−	−	++
RR (breaths/min)	14	18	20	16	18	20	20	20	22	22	22	20	20	20	22	22
SpO_2_/FiO_2_ (ratio)	98/0.21 (467)	98/0.21 (467)	97/0.21 (462)	98/0.21 (467)	98/0.21 (467)	98/0.21 (467)	97/0.21 (462)	98/0.21 (467)	96/0.21 (457)	97/0.21 (462)	94/0.21 (448)	95/0.21 (452)	95/0.21 (452)	96/0.21 (457)	96/0.21 (457)	88/0.21 (419)
HR (beats/min)	82	112	108	124	110	108	96	88	86	86	90	94	72	76	72	82
Glasgow coma scale	E3M1V4	E4V2M6	E4V2M6	E2V2M5	E3V2M5	E3V4M5	E3V4M5	E3V4M5	E3V5M6	E3V5M6	E3V4M5	E3V4M5	E3V4M5	E3V4M5	E3V4M5	E3V4M5
Laboratory findings																
SARS‐CoV‐2 RNA	−	−	−	−	−	−	−	−	−	−	−	−	−	−	−	detected
Hgb (g/dL)	14.4	−	−	13.5	−	−	−	11.4	−	−	−	13.5	−	−	−	13.2
Hct (%)	42.2	−	−	39.0	−	−	−	33.5	−	−	−	39.3	−	−	−	38.8
WBC (cells/mm^3^)	7320	−	−	12270	−	−	−	7520	−	−	−	8580	−	−	−	5260
ANC (cells/mm^3^)	3945	−	−	9767	−	−	−	5866	−	−	−	6735	−	−	−	3840
ALC (cells/mm^3^)	2481	−	−	994	−	−	−	850	−	−	−	892	−	−	−	899
Plts (/mm^3^)	185000	−	−	172000	−	−	−	275000	−	−	−	301000	−	−	−	211000
ALT (U/L)	22	−	−	−	−	−	−	−	−	−	−	−	−	−	−	51
Cr (mg/dL)	0.6	−	0.6	−	−	−	0.8	0.8	0.8	−	0.6	−	−	0.6	0.6	0.7
Management																
Oxygen (L/min)	−	−	−	−	−	−	−	−	−	−	NC (3)	NC (3)	NC (3)	NC (3)	NC (3)	NC (3)
Manidipine	10 mg	10 mg	10 mg	20 mg	20 mg	20 mg	20 mg	20 mg	20 mg	20 mg	20 mg	20 mg	20 mg	20 mg	20 mg	20 mg
Hydralazine	−	−	−	50 mg	50 mg	50 mg	50 mg	50 mg	50 mg	50 mg	50 mg	50 mg	50 mg	50 mg	50 mg	50 mg
Metoprolol	−	−	−	50 mg	100 mg	100 mg	100 mg	100 mg	100 mg	100 mg	100 mg	100 mg	25 mg	25 mg	25 mg	25 mg
Amiodarone	−	−	−	400 mg	400 mg	400 mg	200 mg	200 mg	200 mg	100 mg	−	−	−	−	−	−
Hydroxychloroquine	−	−	−	−	−	−	−	−	−	−	−	−	−	−	−	400 mg q 12 h
Darunavir + Ritonavir	−	−	−	−	−	−	−	−	−	−	−	−	−	−	−	800/100 mg
Favipiravir	−	−	−	−	−	−	−	−	−	−	−	−	−	−	−	1600 mg q 12 h
Azithromycin	−	−	−	−	−	−	−	−	−	−	−	−	−	−	−	500 mg

Abbreviations: ALT, alanine aminotransferase; ALC, absolute lymphocyte count; ANC, absolute neutrophil count; Cr, creatinine; FiO2, fraction of inspired oxygen; Hgb, hemoglobin; Hct, hematocrit; HR, heart rate; hs‐CRP, high sensitivity C‐reactive protein; ICU, intensive care unit; IL‐6, interleukin‐6; LDH, lactate dehydrogenase; NC, nasal cannula; Plts, platelets; RR, respiratory rate; SpO2, arterial oxygen saturation; SOB, shortness of breath; WBC, white blood cell count.

**Figure 4:**
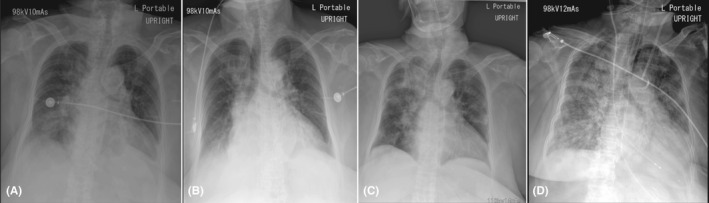
Chest imaging of a 78‐year‐old Thai woman (case 3). A, Chest radiograph on day 3 of hospitalization, showing increased alveolar infiltration in both lower lung zones. B, Chest radiograph on day 5 of hospitalization, showing decreased infiltration in both lower lung zones. C, Chest radiograph on day 15 of hospitalization, showing progression of alveolar infiltration in the right upper, right middle, and right lower lung zones, and in the left upper and left lower lung zones. D, Chest radiograph on day 31 of hospitalization, showing diffuse alveolar infiltration in both lungs with increased right pleural effusion

On day‐ 3 of hospitalization, the patient had fever and atrial fibrillation with rapid ventricular rate of 124 beats/min. Amiodarone was administered for controlling the heart rate and blood pressure within a range of 66‐92 beats/min and 129/68‐164/63 mmHg, respectively. The GCS score decreased to 9 (E2M2V5), but motor power was still grade III on the right extremities. The patient had no cough and no shortness of breath, but lungs showed crepitation in both lower lobes. Chest radiograph showed increased alveolar infiltration in both the lower lung zones (Figure 4A). Laboratory findings showed leucocytosis (12270 cells/mm^3^) with neutrophilia (9767 cells/mm^3^), and urinalysis revealed 30‐50 white blood cells/high power field. Doripenem (500 mg every 8 hours, intravenously) was then given due to suspected hospital‐acquired pneumonia and urinary tract infection. Two samples of hemoculture were negative, but sputum culture showed *Klebsiella pneumoniae*, and urine culture showed *Escherichia coli* >10^5^ CFU/mL. Both organisms were sensitive to ceftriaxone, and the antibiotic was then tapered to ceftriaxone (2 g once daily, intravenously). Between days‐ 5 and 9 of hospitalization, the patient had no fever, and chest radiograph showed decreased infiltration in both lower lung zones (Figure 4B).

On day‐ 10 of hospitalization, the patient had no fever, but she had fatigue, watery diarrhea, increased respiratory rate (22 breaths/min), decreased SpO_2_ (94%), and lung crepitation at the right upper lobe (Table 3). Chest radiograph showed alveolar infiltration at the right upper lung zone and interstitial infiltration at the left lower lung zone. Complete blood analysis showed normal white blood cell count, but low ALC (892 cells/mm^3^).

On day‐ 13 of hospitalization, the patient had dry cough, fatigue, and watery diarrhea (Table 3). Chest radiograph showed increased alveolar infiltration at the right upper, middle, and lower lung zones, but no change in the interstitial infiltration at the left upper and lower lung zones. Sputum smears for acid‐fast bacilli were negative for 3 consecutive days.

On day‐ 15 of hospitalization, the patient still had dry cough, fatigue, and watery diarrhea, but shortness of breath with decreased SpO2 (88% in room air) developed (Table 3). She had a contact history with four family members with confirmed COVID‐19, but the time elapsed from exposure to symptoms was unknown. Chest radiograph showed progression of alveolar infiltration at the right upper, middle, and lower lung zones and at the left upper and lower lung zones (Figure 4C). Both nasopharyngeal and throat swab samples were then tested for SARS‐CoV‐2 RNA using RT‐PCR. The results showed detectable SARS‐CoV‐2 RNA levels at the region of ORF1ab, N gene, NS‐1, and NS‐2 in both samples. Laboratory findings showed normal white blood cell count with low ALC (899 cells/mm^3^) and mildly elevated ALT levels (51 U/L). The patient was transferred to the negative pressure isolation ward and received antiviral treatment with hydroxychloroquine (400 mg every 12 hours for 1 day, then 200 mg every 12 hours, orally), darunavir plus ritonavir (800/100 mg once daily, orally), and favipiravir (1600 mg every 12 hours for 1 day then 600 mg every 12 hours, orally). Azithromycin (500 mg then 250 mg once daily, orally) was given for anti‐inflammation.

Between days‐ 16 and 17 of hospitalization, the patient had low‐grade fever (37.5‐38.2°C), cough, fatigue, watery diarrhea, and shortness of breath (Table 4). She was then transferred to the ICU with negative pressure isolation and received supplement oxygen (3 L/min) via a nasal cannula while in the prone position, in order to keep SpO_2_ >94%. On day‐ 18 of hospitalization, the patient had shortness of breath with decreased SpO_2_ (90% in room air) and received endotracheal tube intubation with ventilator support due to hypoxic respiratory failure. Chest radiograph showed increased alveolar infiltration and pleural effusion in both lungs. On day‐ 19 of hospitalization, she had no fever but still had dry cough and worsening hypoxemia, with a low SpO_2_/FiO_2_ ratio (98), determining the occurrence of severe ARDS. Laboratory findings showed a low ALC (619 cells/mm^3^) and increased serum creatinine levels (>0.3 mg/dL) from baseline, determining the diagnosis of acute kidney injury (AKI) (Table 4).

**Table 4:** Symptoms and signs, laboratory findings, and management of a 78‐year‐old Thai woman (case 3) between day 16 and day 31 of hospitalization

Day of hospitalization	16 (ICU)	17 (ICU)	18 (ICU)	19 (ICU)	20 (ICU)	21 (ICU)	22 (ICU)	23 (ICU)	24 (ICU)	25 (ICU)	26 (ICU)	27 (ICU)	28 (ICU)	29 (ICU)	30 (ICU)	31 (ICU)
Symptoms and signs																
Fever (ºC)	37.5	38.2	37.2	36.5	35.8	36.0	36.3	35.7	35.0	35.0	35.0	35.0	35.0	35.0	35.0	35.0
Cough	+	+	+	+	+	+	+	+	+	+	+	+	+	+	+	+
Fatigue	+	+	+	NA	NA	NA	NA	NA	NA	NA	NA	NA	NA	NA	NA	NA
Diarrhea	++	++	++	++	++	+	+	++	++	+	−	−	+	−	−	−
Dyspnoea	++	++	++	++	++	++	++	+++	++	++	+++	+++	+++	+++	+++	+++
RR (breaths/min)	24	26	24	28	28	28	28	30	24	24	28	30	32	32	30	40
SpO_2_/FiO_2_ (ratio)	95/0.21 (452)	93/0.21 (443)	90/0.21 (428)	88/0.9 (98)	91/0.9 (101)	94/0.9 (104)	88/0.9 (98)	85/0.9 (94)	94/0.8 (118)	94/1.0 (94)	83/1.0 (83)	91/1.0 (91)	94/0.7 (134)	92/0.9 (102)	93/0.9 (103)	91/0.9 (101)
HR (beats/min)	106	92	82	74	68	74	68	76	64	48	32	76	78	76	78	78
Glasgow coma scale	E3V5M6	E3V5M6	E2V5M6	E1VTM5	E1VTM5	E1VTM4	E1VTM4	E1VTM4	E1VTM1	E1VTM1	E1VTM1	E1VTM1	E1VTM1	E1VTM1	E1VTM1	E1VTM1
Laboratory findings																
SARS‐CoV‐2 RNA	−	−	−	−	−	−	−	−	−	−	−	−	−	−	undetected	−
Hgb (g/dL)	−	−	−	11.7	−	−	−	11.1	11.8	−	12.9	10.7	8.3	11.3	11.5	11.8
Hct (%)	−	−	−	34.3	−	−	−	32.5	34.9	−	37.4	32.0	24.5	32.5	32.0	34.0
WBC (cells/mm^3^)	−	−	−	8720	−	−	−	8220	6930	−	9530	15030	13590	19650	32160	42890
ANC (cells/mm^3^)	−	−	−	7534	−	−	−	6896	5696	−	8853	14263	12204	18058	27658	32168
ALC (cells/mm^3^)	−	−	−	619	−	−	−	468	457	−	200	346	557	609	772	3431
Plts (/mm^3^)	−	−	−	280000	−	−	−	229000	190000	−	204000	50000	107000	129000	103000	129000
hs‐CRP (mg/L)	−	−	−	−	273.8	−	−	−	−	−	228.0	168.0	−	−	32.0	20.3
IL‐6 (pg/mL)	−	−	−	−	−	−	−	−	−	−	358.0	2678.0	−	−	−	−
ALT (U/L)	−	−	−	−	44	−	40	42	43	110	−	−	−	−	−	−
Cr (mg/dL)	−	−	−	1.04	1.26	1.22	1.21	1.46	−	1.95	2.29	2.67	1.50	−	−	0.73
Procalcitonin (ng/mL)	−	−	−	−	−	−	−	−	−	−	−	0.87	−	−	−	−
Lactate (mmol/L)	−	−	−	−	−	−	−	−	−	−	4.5	3.4	−	−	−	9.1
Management																
Oxygen supplement (L/min)	NC (3)	NC (3)	NC (3)	MV	MV	MV	MV	MV	MV	MV	MV	MV	MV	MV	MV	MV
Manidipine	20 mg	−	−	−	−	−	−	−	−	−	−	−	−	−	−	−
Hydralazine	50 mg	37.5 mg	−	−	−	−	−	−	−	−	−	−	−	−	−	
Metoprolol	25 mg	25 mg	−	−	−	−	−	−	−	−	−	−	−	−	−	−
Hydroxychloroquine	200 mg q 12 h	200 mg q 12 h	200 mg q 12 h	200 mg q 12 h	200 mg q 12 h	200 mg q 12 h	200 mg q 12 h	200 mg q 12 h	200 mg q 12 h	−	−	−	−	−	−	−
Darunavir + Ritonavir	800/100 mg	800/100 mg	800/100 mg	800/100 mg	800/100 mg	800/100 mg	800/100 mg	800/100 mg	800/100 mg	−	−	−	−	−	−	−
Favipiravir	600 mg q 12 h	600 mg q 12 h	600 mg q 12 h	600 mg q 12 h	600 mg q 12 h	600 mg q 12 h	600 mg q 12 h	600 mg q 12 h	600 mg q 12 h	600 mg q 12 h	600 mg q 12 h	600 mg q 12 h	600 mg q 12 h	−	−	−
Azithromycin	250 mg	250 mg	250 mg	250 mg	250 mg	250 mg	250 mg	250 mg	250 mg	250 mg	250 mg	250 mg	250 mg	−	−	−
Tocilizumab	−	−	−	−	−	−	−	−	−	−	560 mg	−	−	−	−	−

Abbreviations: ALT, alanine aminotransferase; ALC, absolute lymphocyte count; ANC, absolute neutrophil count; Cr, creatinine; FiO2, fraction of inspired oxygen; Hgb, hemoglobin; Hct, hematocrit; HR, heart rate; hs‐CRP, high sensitivity C‐reactive protein; ICU, intensive care unit; IL‐6, interleukin‐6; LDH, lactate dehydrogenase; MV, mechanical ventilation; NC, nasal cannula; Plts, platelets; RR, respiratory rate; SpO2, arterial oxygen saturation; SOB, shortness of breath; WBC, white blood cell count.

**Figure 5:**
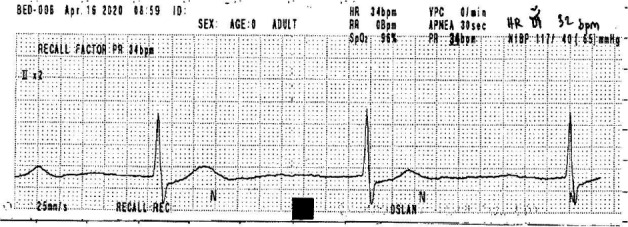
Electrocardiography on day 26 of hospitalization, of a 78‐y‐old Thai woman (case 3), showing bradycardia, with a heart rate of 32 beats/min due to sick sinus syndrome

On day‐ 26 of hospitalization (Table 4), the patient developed shock, as blood pressure was 63/32 mmHg, and the mean arterial pressure (MAP) was 42 mm Hg. Norepinephrine was administered intravenously to keep MAP >65 mm Hg. The patient developed bradycardia with a heart rate of 32 beats/min due to sick sinus syndrome (Figure 5) and was then on a temporary pacemaker at a pace rate of 70 beats/min; dexamethasone (4 g every 8 hours, intravenously) was administered due to suspected myocarditis. Chest radiograph showed decreased infiltration in the left upper lung zone, but no change in both the lower and right upper lung zone infiltration. Right basal pleural effusion was slightly decreased. The hs‐CRP levels were still high (228.0 mg/L), and IL‐6 levels were high (358.0 pg/mL). Tocilizumab (8 mg/kg/dose intravenous drip for 90 minutes) was then administered in order to attenuate lung inflammation. Other laboratory findings showed high arterial blood lactate (4.5 mmol/L) and increased serum creatinine levels (2.29 mg/dL). Meropenem (1 g every 24 hours, intravenously) was administered for suspected hospital‐acquired infection.

On day‐ 27 of hospitalization (Table 4), the patient had low body temperature of 35.0°C, but had productive cough with yellow phlegm, watery diarrhea, and shortness of breath. Chest radiograph showed interstitial infiltration at both lungs. Laboratory findings showed leucocytosis with neutrophilia and high serum procalcitonin (0.87 ng/mL), but decreased hs‐CRP (168.0 mg/L) and arterial blood lactate (3.4 mmol/L) levels. Serum creatinine levels remained increased (2.67 mg/dL), and continuous veno‐venous hemofiltration (CVVH) was then started, indicated by fluid overload.

On day‐ 30 of hospitalization, the patient still had shock (Table 4). Laboratory findings showed marked leucocytosis and decreased hs‐CRP levels (32.0 mg/L). However, SARS‐CoV‐2 RNA detection using RT‐PCR assay was negative on two repeated samples of nasopharyngeal and oropharyngeal swabs. On day‐ 31 of hospitalization, the chest radiograph showed diffuse alveolar infiltration in both lungs with increased right pleural effusion (Figure 4D).Vancomycin (1 g every 48 hours, intravenously) and micafungin (100 mg every 24 hours, intravenously) were added for septic shock and presence of immunosuppression, but her clinical status did not improve and she died on day‐ 31 of hospitalization.

## 3 DISCUSSION

We reported three cases of patients with confirmed SARS‐CoV‐2 infection with severe pneumonia according to the definition of WHO,^12^ characterized by fever, cough, dyspnoea, bilateral lung infiltration, and SpO_2_ <94% in room air. All patients had chronic medical illness, shown to be more common among patients with severe pneumonia (38.7%) compared to those without (21.0%).^4^ There was one older patient (case 3) who died after developing severe ARDS, characterized by arterial oxygen tension/FiO_2_ ≤100 mm Hg with positive end‐expiratory pressure ≥5 cm H_2_O, septic shock, sick sinus syndrome, and AKI. A previous study showed that older patients with confirmed SARS‐CoV‐2 infection and aged ≥65 years are at risk of developing ARDS (hazard ratio 3.26) and of progressing from ARDS to death (hazard ratio 6.17).^13^

Regarding exposure history, two of the patients (cases 1 and 2) were healthcare workers and had a history of exposure to a patient with COVID‐19, with a time to illness development after exposure of 15 days in case 1 and 17 days in case 2. The time elapsed from exposure to symptoms was unknown in case 3. A previous study showed a median incubation period of 4 days among patients with COVID‐19 pneumonia, which was shorter than that among our patients.^4^

The duration from the onset of symptoms to the development of pneumonia was only a few days: 3 days in case 1 and 2 days in case 2. Severe pneumonia, defined according to WHO,^12^ developed on day 6 of illness in cases 1 and 3 and on day 8 in case 2. However, a previous report showed a median time from symptom onset to pneumonia development of 5 days and to severe pneumonia development of 13 days, both longer than those in our report.^7^ Chest CT scan in cases 1 and 2 showed ground‐glass opacity with local patchy shadowing. However, chest radiograph was normal in case 1 and showed minimal local patchy shadowing in case 2. In addition, chest radiograph in case 3 showed local patchy shadowing with interstitial abnormality. Similarly, a previous study showed abnormalities on chest radiograph in 59.1% of patients with COVID‐19, which were fewer than abnormalities on chest CT scan observed in 86.2% of the patients^4^; these abnormalities included ground‐glass opacity (56.4%), bilateral patchy shadowing (51.8%), local patchy shadowing (41.9%), and interstitial abnormalities (14.7%).^4^

Laboratory findings in our patients showed normal white blood count with lymphopenia (ALC <1500 cells/mm^3^). A previous study reported that lymphopenia was present in 83.2% of patients with confirmed SARS‐CoV‐2 infection, and particularly in those with severe pneumonia (96.1%), those admitted to ICU, used mechanical ventilation, or died (92.6%).^4^ The levels of hs‐CRP, LDH, and IL‐6 were increased in our patients, consistent to what was previously reported among patients with severe pneumonia.^4^, ^14^

With the development of severe pneumonia, which was refractory to a combination antiviral therapy, including hydroxychloroquine combined with lopinavir plus ritonavir and favipiravir in cases 1 and 2, slow infusion of 8 mg/kg/dose tocilizumab for 90 min, with a repeated dose in the next 12 hours, was performed in order to attenuate lung inflammation. Both patients were admitted to the ICU and required supplemental oxygen therapy in the prone position in order to reach the target of SpO_2_ >94%. The symptoms of both patients improved, as indicated by the rapid resolution of fever and decreased oxygen supplementation within 24 hours. The levels of hs‐CRP rapidly decreased, lung opacities also decreased, and IL‐6 level increased after receiving tocilizumab. This may be due to the decrease function of IL‐6 as a critical mediator for the development of cytokine storms, leading to respiratory failure, shock, and organ dysfunction in patients with severe COVID‐19 pneumonia.^4^, ^5^, ^6^

However, our third patient (case 3) developed severe ARDS and one dose of tocilizumab (8 mg/kg/dose for 90 min) was administered with dexamethasone (12 mg daily). After receiving treatments with tocilizumab and the corticosteroid, his hs‐CRP levels were still high, and IL‐6 levels increased. The patient died due to cobacterial infection within 2 weeks after ICU admission. A previous study showed common complications among patients with severe COVID‐19 pneumonia, including ARDS (15.6%), septic shock (6.4%), and AKI (2.9%),^4^ which was similar to what we observed in case 3.

No clinical trial has been carried out on the effectiveness of a combination antiviral therapy. However, several studies have been conducted on pharmacological treatments with potential clinical benefit and adjunctive pharmacological treatments.^15^ After a complete 14‐day treatment course of favipiravir in two of our patients, SARS‐CoV‐2 RNA was undetectable within 1 day and 2 days in cases 2 and 3, respectively; however, SARS‐CoV‐2 RNA was still detectable for at least 36 days in case 1 who had a complete 10‐day treatment course of favipiravir. Similarly, a previous study showed favipiravir as an independent factor associated with faster viral clearance than that with lopinavir plus ritonavir (hazard ratio 3.43).^9^

A systematic review showed that tocilizumab may be used as a potential adjunctive pharmacological treatment for patients with COVID‐19, particularly for those with high IL‐6 levels.^11^ A previous study showed that tocilizumab dose ranged from 80 to 600 mg, with an average of 1.5 doses among 15 patients with COVID‐19, 47% of whom were critically ill; the case fatality rate was 20%.^16^ Further ongoing randomized controlled trials may help to clarify the benefit of tocilizumab for COVID‐19 particularly severe COVID‐19 pneumonia.^11^

## 4 CONCLUSION

Early diagnosis and timely treatment of severe COVID‐19 pneumonia may be important for the survival of patients to prevent the development of a cytokine storm, which is a critical mediator of respiratory failure, shock, and organ dysfunction. Tocilizumab could be used in patients with severe COVID‐19 pneumonia who did not respond to a combination of antiviral therapy. However, the use of a combination antiviral therapy should be further confirmed in clinical trials or national regulations with close monitoring.

## 5 INFORMED CONSENT

Informed consent was obtained from the patients.

## CONFLICT OF INTEREST

None declared.

## AUTHOR CONTRIBUTIONS

MS, VT, DS, and VD: performed literature review. MS, YK, SK, TR, WL, SH, SJ, and SL: conceived study and patients’ care. MS, VT, SC, DS, and VD: analyzed data. MS, YK, VT, SK, SC, TR, WL, DS, SH, SJ, SL, and VD: contributed to the writing of the manuscript.

## Data Availability

The authors confirm that the data supporting the findings of this study are available within the article.
